# Changing Paradigms in Antibiotic Resistance in *Salmonella* Species with Focus on Fluoroquinolone Resistance: A 5-Year Retrospective Study of Enteric Fever in a Tertiary Care Hospital in Kolkata, India

**DOI:** 10.3390/antibiotics11101308

**Published:** 2022-09-26

**Authors:** Malabika Biswas, Silpak Biswas, Bishal Gupta, Maria Teresa Mascellino, Anindita Rakshit, Banya Chakraborty

**Affiliations:** 1 Department of Microbiology, School of Tropical Medicine, Kolkata 700073, India; 2 Department of Microbiology, Sanjay Gandhi Post Graduate Institute of Medical Sciences, Lucknow 226014, India; 3 Department of Public Health and Infectious Diseases, Sapienza University of Rome, 00185 Rome, Italy

**Keywords:** *Salmonella*, antibiotics, fluoroquinolones, blood culture, public health

## Abstract

Enteric fever, a potentially fatal multisystem disease that is caused by *Salmonella enterica* serovar Typhi and Paratyphi, poses a significant risk in low- and middle-income countries. A retrospective study to understand the prevalence and evolving patterns of antibiotic resistance in *Salmonella* Typhi and Paratyphi was undertaken from June 2017 to June 2022. A total of 4051 blood samples were collected from patients attending inpatient and outpatient departments of the School of Tropical Medicine (Kolkata, India) hospital. Blood samples were cultured, and culture positive samples were further processed for identification using conventional and automated systems. Antibiotic susceptibility test was performed using both the Kirby-Bauer disc diffusion method and VITEK2 (bioMerieux). Forty-five (1.1%) *Salmonella* species were isolated among the number of total (*n* = 4051) samples that were tested. Out of the 45 *Salmonella* isolates, 35 were *Salmonella* Typhi (77.77%) and 10 were *Salmonella* Paratyphi A (22.23%). We found pronounced fluoroquinolone resistance of 100% in the recent years (2019–2022) in both of the *S*. Typhi and *S*. Paratyphi A isolates. We found that 1 *Salmonella* Typhi and 2 *Salmonella* Paratyphi A isolates were resistant against multiple antibiotics (cefixime, ceftriaxone, ciprofloxacin and nalidixic acid), and 1 multidrug-resistant (MDR) *Salmonella* Paratyphi A isolate was found in a recent study year (2020) and it showed resistance against different classes of antibiotics (cephalosporins, fluoroquinolones and carbapenems). There was no resistance that was detected to the 3^rd^ generation cephalosporins in the final years of the study. The emergence of *Salmonella* isolates that are resistant to multiple antibiotics poses a serious health problem. The antimicrobial resistance patterns that were detected in the study thus warrant further studies to understand the antibiotic susceptibility and resistance pattern of *Salmonella* against the major classes of antibiotics.

## 1. Introduction

Typhoid is an invasive bacterial infection that is caused by the Gram-negative bacteria *Salmonella enterica* subspecies *enterica* serovars Typhi (*Salmonella* Typhi) and Paratyphi A, B and C (known as typhoidal *Salmonella*) [1]. The transmission occurs mainly through contaminated food and water [2].

Enteric fever is a very important cause of undifferentiated acute febrile illness. There are variations among the etiology of different febrile illnesses according to geographic location, age, seasonality and the availability of a testing panel [3,4,5]. There is an estimated >14 million cases of enteric fever annually, and >135,000 deaths that are affecting mostly children and young adults [2]. Enteric fever is endemic in all parts of India, and it still constitutes a major health concern. Almost 30% of the community-acquired blood stream infections in Asia were due to *Salmonella* Typhi [6]. *Salmonella* Paratyphi A is an emerging pathogen in Asia that causes up to 35% of the enteric fever episodes in India and Nepal [7]. It should be noted that as a disease entity, paratyphoid fever cannot be clinically differentiated from typhoid [8].

As is the case for many bacterial infections, there is no test to reliably diagnose enteric fever. The gold standard for diagnosis remains a blood culture which is a slow method, as well as an expensive method. It takes up to 4 days to identify the causative organism and analyze their susceptibility profiles due to low-grade bacteremia [9,10]. Antibiotic susceptibility testing (AST) plays a major role in predicting the local susceptibility patterns [11]. The relevant expertise and facilities that are needed to provide this aid are still deficient in most of the low- and middle-income countries (LMIC) [11]. In such countries, patients may not have access to health facilities, or a blood culture may not be performed as each test incurs further expenses for the patient [12].

Clinicians initiate empirical antibiotic therapy on the basis of their clinical judgment. This presumptive treatment of the disease has likely influenced the antimicrobial patterns in *Salmonella* Typhi [13]. South and South-East Asia constitute critical hubs for enteric fever [14]. The current recommendation for treatment of enteric fever by WHO is chloramphenicol, ampicillin, cotrimoxazole, fluoroquinolones, third-generation cephalosporins (ceftriaxone, cefixime) and azithromycin [9]. Traditionally, MDR *Salmonella* Typhi is used to describe a disease that has combined resistance to chloramphenicol, cotrimoxazole and ampicillin [15]. This has led to fluoroquinolones (FQ) being adopted as the treatment of choice by the late 1990s [16]. These antibiotics were highly effective, and they could be administered orally along with fewer side effects and with rapid bacteraemic clearance [16]. Ciprofloxacin and ofloxacin replaced the first-line antibiotics; however, fluoroquinolone resistance began to develop, which was characterized by a mutation in the fluoroquinolone target genes [17]. Consequently, *Salmonella* Typhi and *Salmonella* Paratyphi are listed as WHO priority pathogens for Antimicrobial resistance (AMR) surveillance [9,18].

Antibiotic-resistant *Salmonella* outbreaks in various parts across India have been reported since the 1960s [19]. Various systematic reviews have shown incomplete AST reporting and a lack of data in low- and middle-income countries [20,21]. Thus, our study focuses on the understanding of antimicrobial susceptibility and resistance patterns against the major classes of antibiotics that are used in enteric fever treatment in a tertiary care hospital (School of Tropical Medicine) in Kolkata, India in order to assess the lacuna of antibiotic therapy. The School of Tropical Medicine is one of the seven institutions around the world that is exclusively dedicated to the research, care and cure of tropical diseases. The hospital section of the institute known as the Carmichael Hospital for Tropical Diseases and it deals with the investigation and treatment of tropical diseases like malaria, enteric fever, leptospirosis, HIV, melioidosis, brucellosis, scrub typhus, among others. It is a 162-bedded hospital that is providing care to almost exclusively adult patients. In 2006, in the North 24 Parganas district in the State of West Bengal, India, the incidence of typhoid fever was found to be 124 per 100000, which was three times more than it was in 2001. On April 2007, a municipality in the suburban area of Kolkata (the capital of West Bengal) reported an increase in the number of cases which were reported passively from a slum. Blood for culture and serological tests were sent to the Calcutta School of Tropical Medicine. Sixty-five of the one hundred and three suspected patients were seropositive after a Widal test (≥1:80), while one culture was positive for *Salmonella* Typhi [22]. The aim of this study was to understand the prevalence and evolving patterns of antibiotic resistance in *Salmonella* Typhi and *Salmonella* Paratyphi that have occurred for over half a decade.

## 2. Results

In this study, a total of 4051 blood samples were tested, of which, 45 *Salmonella* spp. (1.1%) were isolated. Three hundred and thirty-four samples were tested in the year 2017 and two *Salmonella* spp. were isolated from the tested samples. In 2018, 12 *Salmonella* spp. were isolated from the 681 tested samples. Maximum numbers of *Salmonella* spp. (*n* = 15) were isolated in 2019 from the 938 samples. In the years 2020, 2021 and 2022, we obtained nine (out of 702 tested samples), four (out of 996 tested samples), and three (out of 400 tested samples) *Salmonella* spp., respectively (Table 1). It is evident that the maximum percentage positivity for *Salmonella* spp. was in the year 2018 (1.7%) which declined to 0.4% in the year 2021, and then we saw a marginal rise in this in the year 2022 (0.75%). Table 1 shows the number of *Salmonella* spp. that were isolated against the number of samples that were tested in each year from 2017 to 2022, along with the percentage of positivity.

The maximum preponderance was seen in the age group 21–30 years (*n* = 18, 40%), which was followed by age group 15–20 years (*n* = 14, 31.1%) (Table 2).

Interestingly, among all of the samples that were tested between 2017 and 2022, we obtained *Salmonella* species from 66.67% (*n* = 30) of the males and *Salmonella* species from 33.33% of the females (*n* = 15).

**Figure 1:**
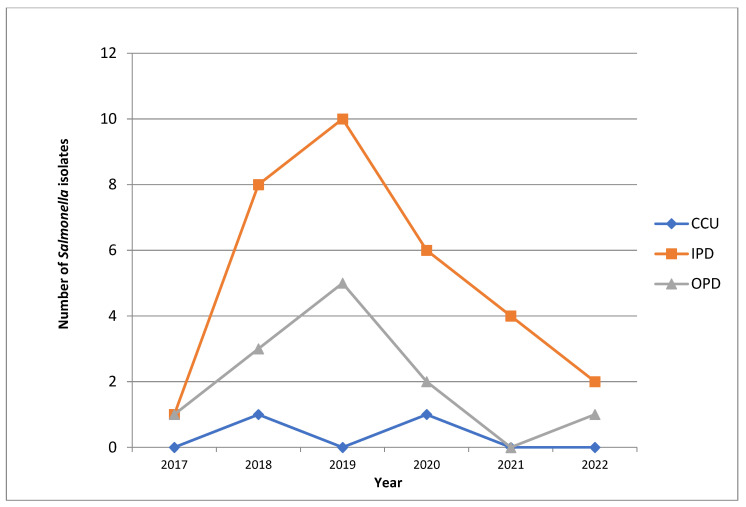
Line diagram showing the annual trends of obtaining *Salmonella* isolates from patients with enteric fever in the OPD, IPD and CCU. OPD: Outpatient Department, IPD: Inpatient Department, CCU: Critical Care Unit.

We also found that a maximum number of patients that were suffering from enteric fever were inpatients (*n* = 30, 67%), in contrast to there being only 13 patients (29%) presenting with the same condition in the outpatient department. Only two patients (4%) were admitted to the critical care unit (Figure 1).

We found an interesting pattern of obtaining *Salmonella* isolates while working on the samples from the OPD, the IPD and the CCU. We isolated a maximum number of *Salmonella* species in the year 2019 (*n* = 15), with the majority being from the inpatient department (*n* = 10), which is in contrast to other years of study where significantly lower numbers of the same condition were isolated (12 in 2018 followed by nine in 2020, four in 2021 and only two in 2017). Figure 1 shows the frequency of obtaining *Salmonella* isolates from patients with enteric fever in the hospital (School of Tropical Medicine, Kolkata, India) and the annual trend of patients in the outpatient department (OPD), the inpatient department (IPD) and the critical care unit (CCU) in the hospital.

In this study, a maximum number of samples were tested in the year 2021, and the highest number of *Salmonella* spp. were isolated in the year 2019 (Table 1). Among the total number of isolates that were found in this study, 77.77% (*n* = 35) were *Salmonella* Typhi, which was followed by *Salmonella* Paratyphi A (*n* = 10, 22.23%). Table 3 shows the number of *Salmonella* Typhi and *Salmonella* Paratyphi A isolates that were obtained in each year from June 2017 to June 2022.

### Antibiotic Resistance Patterns

In the initial year of the study, no resistance to ciprofloxacin and nalidixic acid was found among the *Salmonella* isolates with an average MIC of less <0.25 µg/mL in ciprofloxacin. During 2018, 45.5% of the *Salmonella* isolates showed a resistance to the fluoroquinolones with there being an average MIC of 1 µg/mL. However, from 2019 and onwards, 100% resistance to fluoroquinolones was found among the *Salmonella* isolates with there being an average MIC of 2 µg/mL in the case of ciprofloxacin and ≥32 µg/mL in case of nalidixic acid. Table 4 shows the overall display of antibiotic resistance of 80% of the *Salmonella* Typhi isolates and 90% of the *Salmonella* Paratyphi A isolates to fluoroquinolone compounds, as well as the percentage of resistance of the *Salmonella* isolates to other clinically important antibiotics that are mentioned in this study.

The zone diameters of pefloxacin were compared to both the zone diameters and the MIC of nalidixic acid and ciprofloxacin in order to assess the predictive efficacy of pefloxacin as a surrogate marker of fluoroquinolone resistance. Of the 45 *Salmonella* isolates that were obtained in this study, eight isolates were found to be susceptible to nalidixic acid and ciprofloxacin which were also found to be sensitive to pefloxacin, while 37 isolates that were resistant to nalidixic acid and ciprofloxin were also found to be resistant to pefloxacin. Thus, the positive predictive value of pefloxacin as a surrogate marker of the fluoroquinolones was 100% in our study.

As far as the 3rd generation cephalosporins are concerned, no resistance to either cefixime or ceftriaxone was detected in the initial years of the study (2018–2019). Interestingly, during 2019–2020, we found an increased antibiotic resistance among the *Salmonella* isolates. We observed that the *Salmonella* isolates showed 20% resistance to cefixime (in 2019) and 11.1% resistance to ceftriaxone (in 2020) in total. However, this was not a sustained pattern as no resistance to both of the drugs was detected in the final years of the study (2021–2022). Table 5 shows the percentage of antibiotic resistance that was found in the *Salmonella* Typhi isolates against different antibiotics across 5 years (2017–2022) in this study. A single isolate of *Salmonella* Paratyphi A demonstrated azithromycin resistance. Interestingly, there was a single incidence of meropenem resistance in *Salmonella* Paratyphi A in a patient who was admitted in the critical care unit in the year 2020 (Table 6).

No resistance to ampicillin was detected for any of the *Salmonella* isolates throughout the study. We also found that the bacterial isolates showed no resistance to chloramphenicol in this study. No resistance to azithromycin was noted in all of the *Salmonella* Typhi isolates. Similarly, no resistance was detected to cotrimoxazole in both of the *Salmonella* Typhi and *Salmonella* Paratyphi A through all of the years of the study (Table 5 and Table 6).

Among all the resistant isolates, we found that one *Salmonella* Typhi and two *Salmonella* Paratyphi A isolates showed resistance to multiple antibiotics (cefixime, ciprofloxacin and nalidixic acid). One multidrug-resistant (MDR) *Salmonella* Paratyphi A isolate was also found in this study. The MDR *Salmonella* Paratyphi A isolate showed resistance to different classes of antibiotics such as cefixime (cephalosporins), ceftriaxone (cephalosporins), ciprofloxacin (fluoroquinolones), nalidixic acid (fluoroquinolones) and meropenem (carbapenems).

## 3. Discussion

Multidrug-resistant *Salmonella* spp. have seen an emergence in South-East Asia [23], which is of public health concern. This has been most prevalent against conventional anti-typhoidal drugs such as ampicillin, cotrimoxazole and chloramphenicol. During 1990–1992, the isolates of *Salmonella* Typhi were found to be resistant to chloramphenicol, ampicillin, cotrimoxazole and tetracycline. However, 30–35% of the isolates regained susceptibility to these drugs during 1993–1997 [24]. These developments left few alternatives other than fluoroquinolones to be used as the drug of choice [25,26].

In this present study, the predominant isolate was *Salmonella* Typhi, which was followed by *Salmonella* Paratyphi A. This corroborates with the findings of Manchanda et al. in 2006 [27] and Kumar S et al. in 2008 [28]. The male-to-female ratio in our study was 2:1, which was similar to the studies that were conducted by Chowta et al. [29] who conducted a study of the clinical profile and antibiotic response of typhoid fever in 2005. In high-incidence settings, children appear to bear the brunt of typhoid, but in low-burden settings, the average age of infection increases and in peaks, in some cases, in adolescent and young adult age groups [30,31,32]. This is the case in our study as well, where the maximum prevalence of enteric fever was found to be in the age group 21–30 years (Table 2). We also noted an increase in frequency of patients that were needing hospital admission for enteric fever which was well correlated with the findings of Gupta et al. [33].

In our study, 38 out of 45 (82.2%) *Salmonella* isolates showed fluoroquinolone resistance. Isolates of *Salmonella* Typhi with a reduced susceptibility to fluoroquinolones have been reported previously in the Indian subcontinent [34,35,36]. Interestingly, we found that there was a shift in the fluoroquinolone resistance pattern from 2017 to 2019 (Table 5 and Table 6). This is concordant to the findings of Krishnan et al. [37] who demonstrated a similar decrease in the susceptibility to ciprofloxacin. This can be chiefly attributed to the rise of NARST (nalidixic acid-resistant *Salmonella* Typhi) [38]. There are various factors that are associated with the rise of fluoroquinolone resistance. It may be due to their indiscriminate prescription for the treatment of various infections. It may also be associated with the re-emergence of chloramphenicol susceptibility due to its restricted use, thereby resulting in a withdrawal of the selection pressure [39]. Previous studies have exhibited ciprofloxacin resistance in 21.4% and 18.1% of cases [27,29]. This reduction in fluoroquinolone susceptibility results in a poor clinical response to treatment against the infection that is caused by *Salmonella* spp. Fluoroquinolone resistance is likely to be related to the direct response to an antibiotic pressure, and the uncontrolled use of fluoroquinolones has likely led to the emergence of resistance to this important group of antibiotics. It should be noted that, in concordance to several reports, pefloxacin was found to be a good surrogate marker for ciprofloxacin resistance [40]. Fluoroquinolones exert their antibacterial effects by the inhibition of certain bacterial topoisomerase enzymes, such as DNA gyrase and topoisomerase IV. These are heterotetrameric proteins that are composed of two subunits, which are designated as A and B. The genes encoding the A and B subunits are denoted as *gyrA* and *gyrB* for DNA gyrase or *parC* and *parE* for DNA topoisomerase IV, respectively. A resistance to fluoroquinolones mainly occurs by a mutation in chromosomal genes that encodes the subunits of DNA-gyrase and topoisomerase IV. A small region from codon 67 to 106 of *gyrA* in *E. coli* was designated as the ‘quinolone resistance-determining region’ (QRDR), and variations in this QRDR region were found in species with a natural resistance to fluoroquinolones [41,42,43]. Plasmid-mediated quinolone resistance (PMQR) has also become more frequent and it can spread resistance through horizontal gene transfer [43,44]. Understanding the different mechanisms of the resistance of fluoroquinolone-resistant *Salmonella* isolates at the molecular level is beyond the scope of this study, and further research work on these resistant bacterial isolates will be performed in the near future.

According to the annual report that was published by the Division of Epidemiology and Communicable Diseases, ICMR (January 2021-December 2021) for antimicrobial research network and Surveillance Network, there has been no significant change in the overall antimicrobial susceptibility pattern of *Salmonella* Typhi and *Salmonella* Paratyphi A in India. *Salmonella* Typhi was found to be 100% susceptible to cephalosporins and azithromycin. Drugs such as ampicillin, chloramphenicol and cotrimoxazole also retained a good susceptibility [45].

Fluoroquinolone non-susceptibility (FQNS) is more common in Asia [46], and this has emerged as a major concern in terms of the treatment of typhoid fever. A mathematical modeling study that was supported by Gavi (Gavi, the Vaccine Alliance) and conducted by Birger et al. [46] in 73 countries showed that the baseline total antimicrobial deaths in India was 366,429 [46]. The study also predicted that an introduction of the typhoid conjugate vaccine, including a catch-up campaign for age groups of up to 15 years, could avert an average of 826,000 deaths and 44.4 million DALYs that would be caused by typhoid fever [46]. Vaccination may have an important role in the reduction of enteric fever cases and in decreasing the emergence of antimicrobial resistant strains [47,48]. The two commonly available vaccines of late are an oral vaccine (Ty21a) and the injectable of a Vi polysaccharide vaccine (ViCPS vaccine) [47,48]. The vaccine that was developed by Bharat Biotech, India is more efficacious than the ViCPS vaccine [49]. The vaccine has been registered in India and Nepal but is yet to be implemented across South Asia [50].

Due to the increasing rates of fluoroquinolone resistance, cefixime and ceftriaxone have been adopted as the first line treatment modalities of enteric fever in India [51]. Unfortunately, this has triggered the emergence of extensively drug-resistant (XDR) *Salmonella* Typhi in countries like Pakistan [52]. As far as India is concerned, third generation cephalosporin resistance is associated with presence of AmpC genes *bla*_CMY-2_ [53], *bla*_ACC-1_ [54], *bla*_DHA-1_ [55] and *bla*_SHV-12_ [56]. In India, the prevalence of cephalosporin resistance still remains low, where it ranges from 0% to 5% [55]. A Mumbai-based study that was conducted by Kokare et al. [57] reported ceftriaxone resistance that was as high as 12.5%, which corroborates with our study [57]. Fortunately, we have found that there has been 100% susceptibility to the 3rd generation cephalosporins in the last 2 years. Increasing the MICs for third generation cephalosporins was reported in a few studies that were prior to this one [58,59]. There was the report of a single isolate of carbapenem-resistant *Salmonella* Paratyphi A in our study. A Pakistan-based study in 2020 found that there was 48% resistant to carbapenems in the *Salmonella* isolates [60].

## 4. Materials and Method

### 4.1. Study Type and Study Settings

A retrospective study from laboratory records was performed at the School of Tropical Medicine, Kolkata, India from June 2017 to June 2022.The School of Tropical Medicine, Kolkata, India is a 162-bedded tertiary care hospital.

During the study period, 4051 blood samples were collected from patients that were attending the outpatient department and also admitted in the wards. Relevant data for each patient were collected as well.

### 4.2. Collection of Samples

Blood was collected by sterile, aseptic means. In case of adult patients, 10–15 mL of blood was collected; from the paediatric age group, 5–10 mL of blood was collected.

### 4.3. Processing of Samples

From May 2017 to May 2019, blood was processed using a conventional blood culture medium. The conventional blood culture medium consisted of a brain–heart infusion (BHI) broth. Approximately 10–20 mL of fresh blood was inoculated through the rubber cap of the bottle. The cultures were incubated for 7 days at 37 °C. Repeated subcultures were made on blood agar and MacConkey agar when the broth showed evidence of turbidity.

From June 2019, all of the blood cultures were performed using the BacT/ALERT (bioMerieux System). The BacT/ALERT is an automated blood culture system that uses an internal colorimetric system to detect the presence of carbon dioxide. Growth was continuously monitored, and readings were recorded every 15–20 min. When growth was detected, the system gave a positive signal. Subsequently, the cultures from bottles that were flagged positive were made on blood and MacConkey agar [61].

### 4.4. Identification of Salmonella *spp.*

The identification of *Salmonella* spp. was performed using conventional and automated methods (VITEK 2). For the long-term preservation of strains, the isolates that were confirmed to be *Salmonella* spp. were stored in 15% glycerol broth at −20 °C.

### 4.5. Serotyping of Salmonella *spp.*

Serotyping is a serological procedure that is used to separate the various strains of microorganisms into different groups based on their antigenic composition. The conventional serotyping or antigenic classification of *Salmonella* was based on an antibody reaction with 3 types of surface antigens: O or somatic antigen, H or flagellar antigen and Vi capsular antigen [62]. It should be noted that the antigenic formulae of all *Salmonella* serovars are recorded in the Kauffman-White Le Minor scheme [63]. The O antigen is responsible for the determination of the group of the isolate, while H antigen determines the serovar. The O antigen is a heat-stable polysaccharide that is present on the outer surface lipopolysaccharide [64]. The identification of the O antigen is traditionally carried out in 2 parts: the isolate is tested using an O grouping sera by the process of slide agglutination. Subsequently, the tests are carried out with specific antisera that react with individual antigens [62].

Over the course of our study, the identified *Salmonella* spp. was cultured on nutrient agar. A thick emulsion of an isolated colony was made in normal saline. Both of the polyvalent and monovalent sera were used for serotoyping. For testing with polyvalent sera, one free falling drop of polyvalent O antisera was mixed with the emulsion. The slide was rocked for 30 s and thereafter, it was observed for agglutination. Same procedure was followed for testing with monovalent O antisera.

### 4.6. Antibiotic Susceptibility Testing (AST)

Antibiotic susceptibility testing was performed using both the Kirby Bauer disc diffusion method and VITEK 2 Compact Systems and interpreted according to CLSI guidelines (Clinical Laboratory and Standards Institute) [65]. *Escherichia coli* (ATCC 25922) was used for growth control and for the quality checking of the antibiotic discs. All of the antibiotic discs were procured from HiMedia. The isolates were tested by Kirby Bauer disc diffusion method using ciprofloxacin (5 µg), ceftriaxone (30 µg), chloramphenicol (30 µg), cotrimoxazole (1.25/23.75 µg), piperacillin/tazobactam (100/10), amoxicillin/clavulanic acid (20/10 µg), nalidixic acid (30), cefixime (5 µg), meropenem (10 µg), pefloxacin (5 µg) and azithromycin (15 µg). The *Salmonella* isolates that showed resistance to more than three or more classes of antimicrobial agents were defined as multidrug-resistant (MDR) isolates in this study.

### 4.7. Minimum Inhibitory Concentration (MIC) Determination

The VITEK 2 system was used to perform the testing for the MIC for various antibiotics. In VITEK, the MIC was determined by comparing the growth of the patient isolates to the growth of the isolates with known minimum inhibitory concentrations. This was performed by the continuous monitoring by the machine (biomeriuex, Craponne, France).

## 5. Conclusions

Our study gives an insight into the evolving antibiotic susceptibility and resistance patterns of *Salmonella* spp. that have occurred over the past half-decade. Though there is a pronounced decrease in fluoroquinolone susceptibility, the fact that there is a sustained sensitivity to 3rd generation cephalosporins and chloramphenicol offers us a glimmer of hope. The fluoroquinolone resistance that was found in *Salmonella* Typhi and *Salmonella* Paratyphi A isolates is a public health concern that can lead to treatment failures. The steadily increasing multiple drug resistance in the *Salmonella* isolates is a cause of grave concern in India. This study is of clinical significance, and it also reemphasizes the need for the continuous surveillance and constant monitoring of the antibiotic susceptibility and resistance patterns of *Salmonella* spp. against different clinically relevant antibiotics, and understanding the molecular mechanisms of antibiotic resistance could ultimately help in preventing the salmonellosis in humans.

## Figures and Tables

**Table 1:** The number of *Salmonella* spp. that were isolated against the number of total samples that were tested in each year from 2017 to June 2022, with the percentage of positivity.

Year	Number of Samples Tested	Number of *Salmonella* Isolated	Percentage Positivity (%)
2017	334	2	0.59%
2018	681	12	1.7%
2019	938	15	1.5%
2020	702	9	1.2%
2021	996	4	0.4%
2022	400	3	0.75%

**Table 2:** Frequency of *Salmonella* spp. that were isolated from patients with enteric fever in different age groups from 2017–2022, as found in this study.

	Percentage Positivity across Various Age Groups						
Years	10–15	16–20	21–30	31–40	41–50	51–60	>60
2017	1/30 (3%)	0/7 (0%)	0/67 (0%)	0/62 (0%)	0/74 (0%)	1/46 (2.17%)	0/48 (0%)
2018	0/17 (0%)	4/31 (12.9%)	3/273 (1.09%)	1/104 (0.96%)	2/72 (2.7%)	1/90 (1.1%)	1/94 (1.06%)
2019	2/36 (5.5%)	4/70 (5.7%)	8/332 (2.4%)	0/213 (0%)	0/92 (0%)	0/100 (0%)	1/95 (1.05%)
2020	0/20 (0%)	3/30 (10%)	5/261 (1.9%)	0/91 (0%)	1/153 (0.6%)	0/100 (0%)	0/47 (0%)
2021	0/15 (0%)	2/35 (5.7%)	0/377 (0%)	1/125 (0.8%)	1/101 (0.9%)	0/178 (0%)	0/165 (0%)
2022	0/8 (0%)	1/15 (6.6%)	2/153 (1.3%)	0/100 (0%)	0/85 (0%)	0/14 (0%)	0/25 (0%)

**Table 3:** Number of *Salmonella* Typhi and *Salmonella* Paratyphi A isolates that were obtained in each year from 2017 to 2022.

Year	*Salmonella* Typhi	*Salmonella* Paratyphi A
2017	1	1
2018	11	1
2019	10	5
2020	7	2
2021	3	1
2022	3	0
Total	35	10

**Table 4:** Percentage of resistance of the *Salmonella* isolates to fluoroquinolones and other clinically important antibiotics that are mentioned in this study from 2017 to 2022.

Antibiotics	*Salmonella* Typhi (*n* = 35)	*Salmonella* Paratyphi A (*n* = 10)
Chloramphenicol	0/35 (0%)	0/10 (0%)
Cotrimoxazole	0/35 (0%)	0/10 (0%)
Ampicillin	0/35 (0%)	0/10 (0%)
Ceftriaxone	0/35 (0%)	1/10 (10%)
Azithromycin	0/35 (0%)	1/10 (10%)
Ciprofloxacin	28/35 (80%)	9/10 (90%)
Nalidixic Acid	28/35 (80%)	9/10 (90%)
Pefloxacin	28/35 (80%)	9/10 (90%)
Amoxycillin-Clavulanic Acid	0/35 (0%)	1/10 (10%)
Piperacillin-Tazobactam	0/35 (0%)	1/10 (10%)
Meropenem	0/35 (0%)	1/10 (10%)
Cefixime	1/35 (2.8%)	3/10 (30%)

**Table 5:** Percentages (%) of antibiotic resistance that were found in the *Salmonella* Typhi isolates against different antibiotics across 5 years (2017–2022).

	2017 (*n* = 1)	2018 (*n* = 11)	2019 (*n* = 10)	2020 (*n* = 7)	2021 (*n* = 3)	2022 (*n* = 3)
Ampicillin	0/1 (0%)	0/11 (0%)	0/10 (0%)	0/7 (0%)	0/3 (0%)	0/3 (0%)
Cotrimoxazole	0/1 (0%)	0/11 (0%)	0/10 (0%)	0/7 (0%)	0/3 (0%)	0/3 (0%)
Azithromycin	0/1 (0%)	0/11 (0%)	0/10 (0%)	0/7 (0%)	0/3 (0%)	0/3 (0%)
Chloramphenicol	0/1 (0%)	0/11 (0%)	0/10 (0%)	0/7 (0%)	0/3 (0%)	0/3 (0%)
Ceftriaxone	0/1 (0%)	0/11 (0%)	0/10 (0%)	0/7 (0%)	0/3 (0%)	0/3 (0%)
Cefixime	0/1 (0%)	0/11 (0%)	1/10 (10%)	0/7 (0%)	0/3 (0%)	0/3 (0%)
Ciprofloxacin	0/1 (0%)	5/11 (45.45%)	10/10 (100%)	7/7 (100%)	3/3 (100%)	3/3 (100%)
Nalidixic Acid	0/1 (0%)	5/11 (45.45%)	10/10 (100%)	7/7 (100%)	3/3 (100%)	3/3 (100%)
Pefloxacin	0/1 (0%)	5/11 (45.45%)	10/10 (100%)	7/7 (100%)	3/3 (100%)	3/3 (100%)
Meropenem	0/1 (0%)	0/11 (0%)	0/10 (0%)	0/7 (0%)	0/3 (0%)	0/3 (0%)

**Table 6:** Percentages (%) of antibiotic resistance that were found in the *Salmonella* Paratyphi A isolates against different antibiotics across 5 years (2017–2022).

	2017 (*n* = 1)	2018 (*n* = 1)	2019 (*n* = 5)	2020 (*n* = 2)	2021 (*n* = 1)	2022 (*n* = 0)
Ampicillin	0/1 (0%)	0/1 (0%)	0/5 (0%)	0/2 (0%)	0/1 (0%)	0%
Cotrimoxazole	0/1 (0%)	0/1 (0%)	0/5 (0%)	0/2 (0%)	0/1 (0%)	0%
Azithromycin	0/1 (0%)	0/1 (0%)	0/5 (0%)	1/2 (50%)	0/1 (0%)	0%
Chloramphenicol	0/1 (0%)	0/1 (0%)	0/5 (0%)	0/2 (0%)	0/1 (0%)	0%
Ceftriaxone	0/1 (0%)	0/1 (0%)	1/5 (20%)	1/2 (50%)	0/1 (0%)	0%
Cefixime	0/1 (0%)	0/1 (0%)	2/5 (40%)	1/2 (50%)	0/1 (0%)	0%
Piperacillin/Tazobactam	0/1 (0%)	0/1 (0%)	0/5 (0%)	1/2 (50%)	0/1 (0%)	0%
Amoxicillin/Clavulanic Acid	0/1 (0%)	0/1 (0%)	0/5 (0%)	1/2 (50%)	0/1 (0%)	0%
Ciprofloxacin	1/1 (100%)	0/1 (0%)	5/5 (100%)	2/2 (100%)	1/1 (100%)	0%
Nalidixic Acid	1/1 (100%)	0/1 (0%)	5/5 (100%)	2/2 (100%)	1/1 (100%)	0%
Pefloxacin	1/1 (100%)	0/1 (0%)	5/5 (100%)	2/2 (100%)	1/1 (100%)	0%
Meropenem	0/1 (0%)	0/1 (0%)	0/5 (0%)	1/2 (50%)	0/1 (0%)	0%

## Data Availability

Not applicable.
